# Plant Secondary Metabolites—Central Regulators Against Abiotic and Biotic Stresses

**DOI:** 10.3390/metabo15040276

**Published:** 2025-04-16

**Authors:** Ameer Khan, Farah Kanwal, Sana Ullah, Muhammad Fahad, Leeza Tariq, Muhammad Tanveer Altaf, Asad Riaz, Guoping Zhang

**Affiliations:** 1 Department of Agronomy, College of Agriculture and Biotechnology, Zhejiang University, Zijingang Campus, Hangzhou 310029, China; 12116133@zju.edu.cn; 2 National Key Laboratory for Tropical Crop Breeding, School of Breeding and Multiplication (Sanya Institute of Breeding and Multiplication), Hainan University, Sanya 572025, China; farah@hainanu.edu.au; 3 Department of Plant Breeding and Genetics, University of Agriculture, Faisalabad 38000, Pakistan; sanaullahpbg1@gmail.com; 4 Zhejiang Provincial Key Laboratory of Crop Genetic Resources, College of Agriculture and Biotechnology, Zhejiang University, Hangzhou 310058, China; muhammadfahad@zju.edu.cn; 5 National Key Laboratory for Rice Biology and Breeding, Institute of Biotechnology, Zhejiang University, Hangzhou 310058, China; leeza_tariq@zju.edu.cn; 6 Department of Field Crops, Faculty of Agriculture, Recep Tayyip Erdoğan University, Pazar, Rize 53300, Turkey; muhammadtanveer.altaf@erdogan.edu.tr; 7 Queensland Alliance for Agriculture and Food Innovation, The University of Queensland, Brisbane, QLD 4072, Australia

**Keywords:** abiotic and biotic stresses, defense mechanism, signaling pathway, secondary metabolites, stress tolerance

## Abstract

As global climates shift, plants are increasingly exposed to biotic and abiotic stresses that adversely affect their growth and development, ultimately reducing agricultural productivity. To counter these stresses, plants produce secondary metabolites (SMs), which are critical biochemical and essential compounds that serve as primary defense mechanisms. These diverse compounds, such as alkaloids, flavonoids, phenolic compounds, and nitrogen/sulfur-containing compounds, act as natural protectants against herbivores, pathogens, and oxidative stress. Despite the well-documented protective roles of SMs, the precise mechanisms by which environmental factors modulate their accumulation under different stress conditions are not fully understood. This review provides comprehensive insights into the recent advances in understanding the functions of SMs in plant defense against abiotic and biotic stresses, emphasizing their regulatory networks and biosynthetic pathways. Furthermore, we explored the unique contributions of individual SM classes to stress responses while integrating the findings across the entire spectrum of SM diversity, providing a comprehensive understanding of their roles in plant resilience under multiple stress conditions. Finally, we highlight the emerging strategies for harnessing SMs to improve crop resilience through genetic engineering and present novel solutions to enhance agricultural sustainability in a changing climate.

## 1. Introduction

Plants are constantly subjected to various abiotic and biotic stresses, which severely impact their growth, development, and overall productivity [1]. Abiotic stresses, such as extreme temperatures, drought, salinity, flooding, heavy metals, UV radiation, and pollutants, disrupt essential physiological processes, leading to reduced photosynthesis, impaired water and nutrient absorption, and disordered metabolism [2]. For instance, prolonged drought in regions like sub-Saharan Africa reduces crop yields by impairing water availability, while heavy metal contamination in industrial zones compromises soil health and plant growth. In contrast, biotic stresses arise from interactions with living organisms, including pathogens (e.g., bacteria, viruses, and fungi), pests (e.g., insect herbivores), and competitive weeds. These stressors compromise plant immune defenses, impair nutrient transport, and weaken plant structures, e.g., in rice blast disease (*Magnaporthe oryzae*) or herbivore grazing by pests like the fall armyworm in maize [3,4,5]. As climate change intensifies these challenges, understanding plant resilience mechanisms becomes critical for ensuring food security.

The biochemical co-evolutionary arms-race theory suggests that plant secondary metabolites adapted to herbivore pressure. This has led to the development of herbivore resistance mechanisms over time [6]. Over time, plants and their antagonists have engaged in a dynamic interplay, driving the development of secondary metabolites (SMs) as multifaceted defense tools. For example, plants emit volatile terpenes to deter herbivores and attract predatory insects, while alkaloids exert toxic effects that increase pest mortality or inhibit microbial growth [7]. Additionally, they produce toxic secondary metabolites that enhance herbivore mortality and serve as antibiotic defenses against microorganisms in their innate immune system. These compounds not only protect against immediate threats but also shape ecological interactions, such as recruiting parasitic wasps to control pest populations. This evolutionary perspective underscores SMs as central regulators of plant defense, a theme elaborated across this review’s exploration of their diversity and functions.

To counter both abiotic and biotic stresses, plants have evolved various defense mechanisms to protect themselves. Among these, the modulation of metabolites and metabolic pathways has emerged as a crucial strategy for plant survival and adaptation [8]. Plant metabolites are broadly categorized into two groups: primary metabolites (PMs), such as proteins, carbohydrates, and amino acids, essential for growth, respiration, and reproduction, and SMs, including alkaloids, flavonoids, phenolic acids, and pigments, ubiquitous in plant cells [9,10,11]. Over 200,000 SMs have been identified in plants in recent decades, reflecting their vast chemical diversity and ecological roles [12]. Beyond their well-known applications in medicine, nutrition, and cosmetics, SMs are increasingly recognized in plant sciences for shielding PMs (e.g., nucleic acids and proteins) from stress-induced damage. Under stress, plants activate specific genes to synthesize SMs, enhancing their resilience to adverse conditions like drought or pathogen attack [13]. The role of SMs in stress defense and their genetic regulation have become a focal point in plant stress physiology and molecular biology, underscoring their importance in adaptive resilience [14]. Biotic stressors act as elicitors, stimulating plants to produce secondary metabolites in response to biotic stress. While several recent reviews have explored the roles of secondary metabolites (SMs) in plant stress responses [12], this review distinguishes itself by providing a comprehensive integration of the findings across the full spectrum of SM diversity and multiple stress conditions. In contrast to the previous studies, we emphasized the regulatory networks and biosynthetic pathways underlying SM production, offering a deeper understanding of their roles in plant resilience.

Additionally, we highlight the emerging strategies for leveraging SMs in crop improvement, addressing the gaps in the application of these metabolites in sustainable agriculture. Accordingly, this review aims to consolidate and expand the existing knowledge, providing novel insights into the integral functions of various SMs in supporting plant resilience to abiotic and biotic stresses. Specifically, we explore key groups of protective and adaptive SMs, including alkaloids, flavonoids, phenolic acids, and anti-reactive oxygen species (ROS) enzymes, such as peroxidases, polyphenol oxidases, and chitinases. Furthermore, this review highlights the critical knowledge gaps and proposes future research directions to better harness plant secondary metabolism for developing stress-resilient crop varieties in the face of increasing environmental challenges.

## 2. Diversity of Secondary Metabolites and Their Biosynthesis in Plants

**Figure 1:**
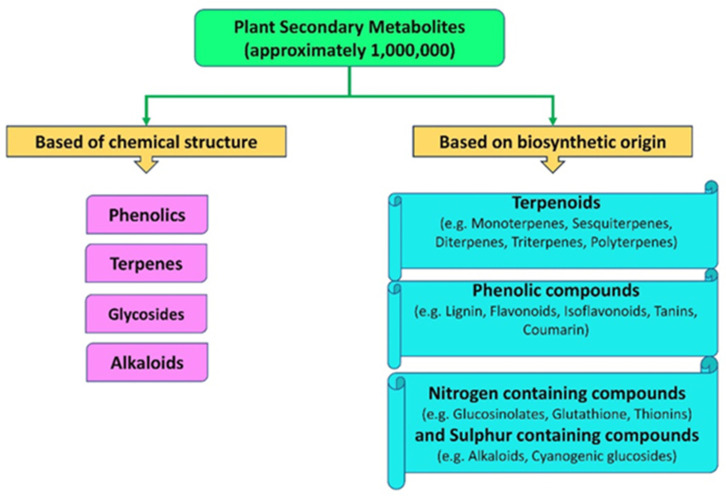
Classification and categories of SMs.

SMs are essential compounds that contribute significantly to plant defense and adaptation against environmental stresses. They are highly diverse and can be classified based on their chemical structures or biosynthetic origins. Structurally, SMs are categorized into four main groups [15]. The first group, phenolics, includes compounds such as phenolic acids, lignin, lignans, tannins, and coumarins, which are widely recognized for their roles as antioxidants and structural components (Figure 1). The second group, terpenes, comprises sterols, volatile compounds, carotenoids, cardiac glycosides, and flavonoids, all of which are crucial for plant signaling, hormonal regulation, and stress response. Nitrogen-containing compounds, the third group, consist of alkaloids and cyanogenic glycosides that typically serve as chemical deterrents against herbivores and pathogens. Lastly, sulfur-containing compounds, such as thionine, lectins, glutathione, defensins, and phytoalexins, are vital in counteracting oxidative stress and enhancing pathogen resistance [12]. Additionally, they strengthen pathogen resistance by disrupting microbial integrity, inhibiting pathogen growth, and activating plant defense responses.

From a biosynthetic perspective, SMs are classified into three primary groups: terpenes, phenolics, and nitrogen- and sulfur-containing compounds. Their biosynthesis occurs through three major pathways: mevalonic acid (MVA), malonic acid, and shikimic acid pathways [16]. These pathways are highly responsive to environmental stress and produce metabolites that enhance plant tolerance. The shikimic acid pathway generates a variety of metabolites, including flavonoids, anthocyanins, tannins, stilbenes, suberin, and lignin [17]. These compounds act as structural barriers to prevent pathogen invasion and as signaling molecules that activate plant defense responses [18]. The methylerythritol phosphate (MEP) pathway, an alternative to the MVA pathway, produces carotenoids, diterpenes, quinones, tocopherols, and gibberellins, which play critical roles in alleviating oxidative damage and modulating hormonal responses under stress conditions [19]. The MVA pathway contributes to the production of sterols that protect plants against oxidative stress and physical damage [20]. Together, these three pathways contribute to the biosynthesis of essential secondary metabolites, including phenolics, terpenoids, and alkaloids, which play key roles in plant defense, growth, and signaling [21]. While the shikimic acid pathway produces aromatic amino acids and phenolics, the MEP and MVA pathways generate terpenoids crucial for hormone synthesis, structural integrity, and stress responses.

The biosynthesis of SMs is intricately linked to plant survival under stress conditions, with the resulting metabolites performing multiple roles. These roles include reinforcing plant structures with compounds like lignin and suberin, neutralizing oxidative damage with antioxidants such as tocopherols and flavonoids, and regulating hormonal responses to maintain physiological balance during stress [22]. In addition to their defensive functions, these metabolites participate in complex signaling networks that enhance plant adaptability. This synergy between biosynthetic pathways and the functional versatility of SMs underscores their central importance in plant stress tolerance and adaptation.

## 3. The Roles of SMs in Plant Stress Responses

**Figure 2:**
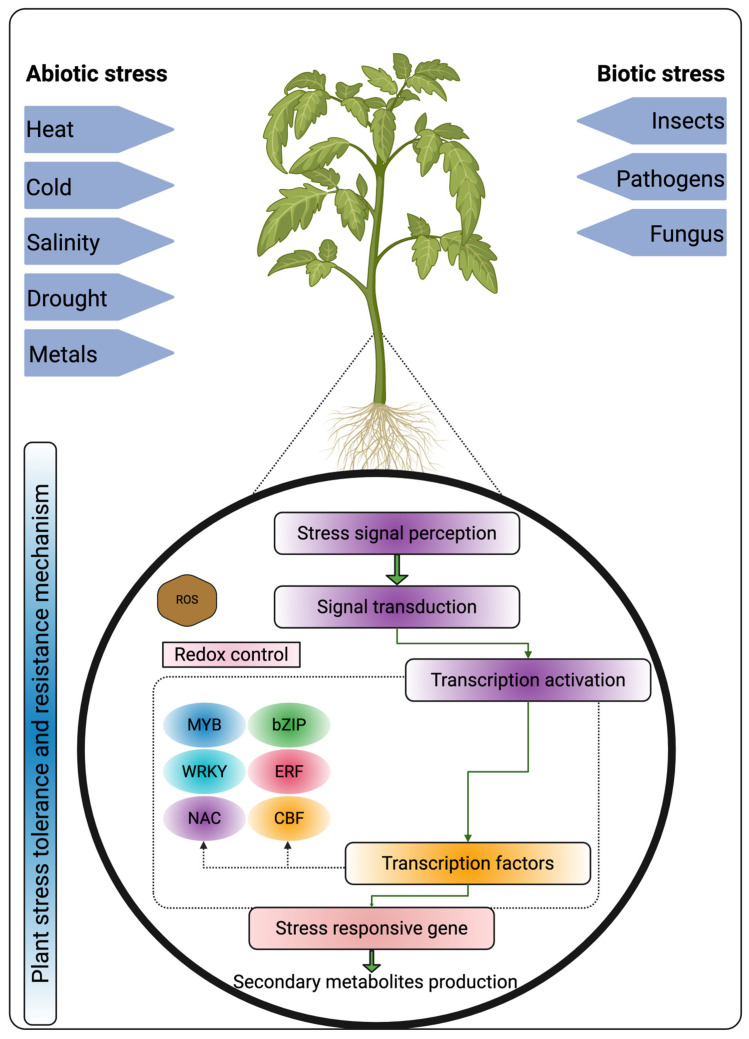
Mechanisms of plant tolerance and resistance to biotic and abiotic stresses. Climate change poses a significant threat to plant survival, particularly in regions vulnerable to extreme conditions. Various ecological stressors, such as insects, pathogens, temperature fluctuations, light intensity, soil salinity, and nutrient availability, significantly impact plant physiological and biochemical responses, including secondary metabolism. Plants produce a range of secondary metabolites as a defense mechanism to mitigate the adverse effects of these stresses.

SMs are vital for plants to develop defense mechanisms and their ability to adapt to environmental stresses. These SMs compounds, often produced in response to biotic and abiotic stresses, act as protective agents against harsh conditions, pathogens, herbivores, and adverse environmental conditions (Table 1; Figure 2) [23]. Each SM group plays distinct roles in plant stress responses. Here, we focused on terpenes, flavonoids, phenolics, tannins, lignans, coumarins, lignin, stilbenes, curcuminoids, chitinases, nitrogen, and sulfur-containing SMs, which play vital roles in plant defense, signaling, and adaptation by acting as antioxidants (flavonoids, phenolics, stilbenes, and curcuminoids), structural components (lignin and lignans), antimicrobial agents (tannins, chitinases, and sulfur- and nitrogen-containing SMs), and growth regulators (terpenes and coumarins) [24].

### 3.1. Terpenes

Plants, being sessile and anchored in their environments, are continuously exposed to challenges from biotic stresses, such as pests and pathogens, and abiotic stresses, including drought, salinity, and extreme temperature. Within this context, terpenes, a diverse class of secondary metabolites (SMs), play critical roles in plant defense and adaptation to both biotic and abiotic stresses. Major crops, such as rice, wheat, maize, and barley, are particularly susceptible to these stressors, which include biotic factors, like insect pests and microbial pathogens, as well as abiotic conditions, such as water deficiency, high salinity, and temperature fluctuations. These stresses collectively contribute to substantial yield reductions, posing significant challenges to agricultural productivity [25]. Rice suffers from bacterial blight, blast disease, and planthoppers, while wheat is affected by rust, Fusarium blight, and aphids; both face drought and heat stress [26]. Maize struggles with fall armyworms and lethal necrosis, whereas barley is prone to powdery mildew and yellow dwarf virus, both experiencing drought and salinity stress [27]. Climate change and pest evolution exacerbate these threats, making stress resistance a critical focus of the agricultural research.

These compounds are synthesized from C5 units, such as dimethylallyl diphosphate (DMAPP) or isopentenyl diphosphate (IPP), and are categorized based on the number of units they contain [28]. Categories include hemiterpenes (C5), monoterpenes (C10), sesquiterpenes (C15), diterpenes (C20), sesterpenes (C25), triterpenes (C30), and polyterpenes (C40) [29]. Therefore, plant compounds, such as terpenes, are actively produced by plants in response to biotic stressors, such as herbivore attacks. For example, *Pinus sylvestris* produces increased amounts of terpenes when subjected to caterpillar feeding, with more terpenes emitted from branches under heavy caterpillar attack than from less-affected branches [30]. These volatile compounds serve as deterrents to herbivores and act as repellents. In addition, transgenic tobacco plants have been shown to release isoprene when attacked by caterpillars, a response that is not observed in wild-type plants [31]. Terpenes also attract pollinators in certain plants, facilitating ecological interactions [8]. Furthermore, sesquiterpenes, lactones, and other compounds, such as taraxinic acid beta-D-glucopyranosyl ester, are known to protect plants from root-feeding pests. For example, dandelions release latex that is rich in terpenes, offering protection from both above-ground and root-feeding stressors [32]. Latex serves a dual purpose, providing a defense mechanism against pathogens and protecting the plant from underground pests [33]. This highlights the need for a deeper understanding of how terpenes interact with multiple layers of plant defense systems.

However, substantial research has been conducted to understand their biochemical pathways and ecological functions, and numerous critical gaps remain in elucidating their exact mechanisms and roles across different plant species and environmental contexts [34]. Terpenes also play an essential role in protecting plants from abiotic stressors, such as drought, salinity, and oxidative stress. For instance, oleuropein production in the leaves and roots of olive trees is a response to salinity stress [35]. This compound helps plants manage oxidative stress resulting from high salt concentrations, demonstrating the protective function of terpenes in saline environments [36]. Oleuropein serves as a glucose reservoir that aids osmoregulation and contributes to plant adaptation to harsh saline conditions [37]. Non-volatile antioxidants, including those in the terpene family, have been identified as vital for enhancing plant stress tolerance. Specific terpenes, such as isoprene, help mitigate the effects of photooxidative stress, ozone stress, and heat stress [38]. Although some plants, such as grapevines, do not emit isoprene, they still produce other terpenoid compounds, such as monoterpenes, which confer heat stress tolerance [39]. Moreover, terpenes are essential for stabilizing plant cell membranes, reducing oxidative stress, and enhancing resilience to abiotic stress.

Compounds such as monoterpene hydrocarbons and isoprene have been shown to possess antioxidant properties, which are crucial for mitigating oxidative damage in response to heat, UV radiation, and other environmental stresses [40]. The interaction of these compounds with ROS-scavenging systems remains crucial for future investigation. Moreover, some acidic terpenoids, including zealexins and kauralexins, also act as phytoalexins that protect plants from pathogens and environmental stressors, including drought and salinity [21]. These terpenoids help maintain biomass production in crops such as maize, although their effectiveness is reduced under severe water deficiency [41]. Similarly, terpenoids, like sabinene, myrcene, and limonene, produced in response to UV-B radiation and hydrogen peroxide, stimulate rice seedling growth and enhance stress resistance [42]. Additionally, certain terpenes, such as carnosic acid, a diterpene, protect plants in the Labiatae family from water stress and other environmental challenges. These findings collectively demonstrate the multifaceted role of terpenes in promoting plant resilience under biotic and abiotic stress conditions. Filling these knowledge gaps is key to advancing our understanding of terpene-mediated plant resilience and improving agricultural practices focused on stress tolerance.

### 3.2. Phenolics

Phenolic compounds are vital for defending plants against biotic stress, including attacks by pests, pathogens, and herbivores [43] These compounds help maintain the structural integrity of the plant by acting as a defense mechanism against harmful agents such as bacteria, fungi, viruses, and nematodes [18]. One prominent group of phenolics, coumarins, is found in plant membranes and plays a key role in plant defense [43]. Coumarins act as antioxidants and antimicrobial agents, scavenging reactive oxygen species (ROS) and inhibiting pathogen growth [44]. They also help with iron mobilization under nutrient stress and enhance plant resilience to drought, salinity, and UV radiation. The accumulation of coumarins enhances their tolerance to fungal, bacterial, and viral infections, which is particularly evident in the defense against pathogens, such as oomycetes [45]. Furthermore, other phenolic compounds, such as ferulic acid and protocatechuic acid, accumulate in rice plants during fungal attacks, reducing the impact of mycotoxins [46]. These findings emphasize the protective roles of phenolic compounds in the face of biotic stress but highlight a gap in our understanding the precise molecular pathways through which phenolics interact with other defense mechanisms.

Phenolic accumulation plays a crucial role in protecting plants from abiotic stresses, such as cold, drought, and heavy metal toxicity [47]. In response to environmental stressors, phenolics contribute to cell wall fortification and antioxidant defense, which stabilize plant structure and protect against oxidative damage [48]. For example, in *Secale cereale* (winter rye), cold stress triggers an increase in phenolic production, specifically the deposition of lignin and suberin, which enhance cell wall stability and cold tolerance [49]. Similarly, phenolic compounds, such as catechin and quercetin, accumulate in corn plants under Al toxicity, helping mitigate oxidative stress [50]. In addition, the production and accumulation of phenolic compounds fluctuate according to environmental conditions, seasonal changes, and plant growth stages [51]. These compounds vary in concentration, increasing or decreasing depending on developmental stage, environmental stress, or pathogen attacks [52]. The varying phenolic concentrations observed during different growth stages or seasonal changes suggest that these compounds play a dynamic role in plant adaptation [53]. Despite their broad protective functions, which include antioxidant, anti-inflammatory, and anti-carcinogenic activities, several critical gaps in our understanding of their synthesis, regulation, and interaction with other plant metabolites remain. Despite these well-established roles, the exact biochemical signaling pathways that regulate phenolic biosynthesis in response to abiotic stresses remain inadequately understood [54]. However, there is limited research on how these fluctuations are regulated at the molecular level. Understanding how phenolic synthesis interacts with plant development and environmental condition will help identify how these compounds can be harnessed to improve plant resilience.

### 3.3. Flavonoids

**Figure 3:**
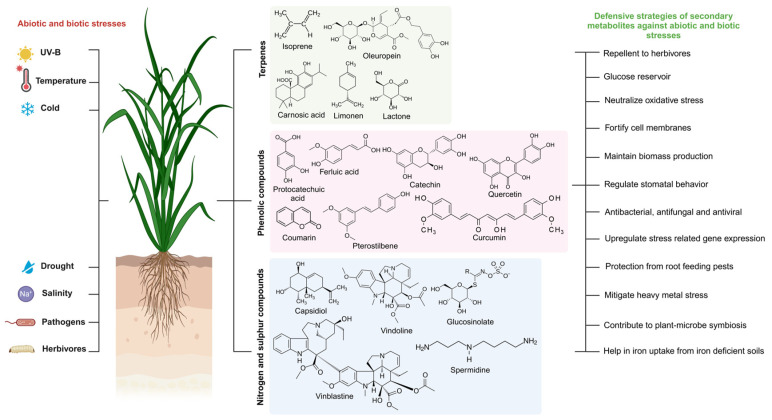
The main pathways producing end products associated with stress tolerance or resistance in plants.

Flavonoids are a diverse class of secondary metabolites that play critical roles in plant defense and stress adaptation. With over 6000 identified structures, flavonoids include anthocyanins, responsible for orange, red, purple, and blue pigmentation, and aurones and chalcones, which contribute yellow colors [55]. These compounds exhibit antioxidant properties and function as phytoalexins, protecting plants against biotic stresses, including insect herbivory and pathogen attacks, and abiotic stresses, such as cold and UV radiation [12]. Flavonoids are synthesized from phenylalanine, a key precursor in the phenylpropanoid pathway, and are characterized by a C6-C3-C6 skeleton consisting of two aromatic rings connected by a heterocyclic ring containing oxygen [56]. This structural diversity enables flavonoids to perform a wide range of functions, including pigmentation, UV protection, and regulation of cell physiology (Figure 3).

Flavonoids act as feeding deterrents for herbivores, such as *Spodoptera exempta*, and serve as biochemical signals that attract beneficial microorganisms and pollinators, facilitating plant–microbe symbiosis [57]. Additionally, they function as phytochemical alexins, combating pathogenic microbes, including bacteria, fungi, and viruses, by regulating reactive oxygen species (ROS) [58] and detoxifying ROS during photosynthetic electron transport [59]. Recent studies have highlighted the antifungal properties of specific flavonoids, such as isoflavones and flavanones, which provide protection against a variety of phytopathogens [60]. Their antimicrobial activity is particularly effective against viral infections, contributing to plant immunity [61]. In summary, flavonoids are essential for plant health, enhancing pathogen-triggered immunity and protecting against a broad spectrum of microbial threats.

Flavonoids also play a pivotal role in abiotic stress responses, such as drought and salinity [62]. Their antioxidant capacity helps mitigate oxidative stress, while their involvement in pigmentation and defense mechanisms enhances plant resilience to environmental challenges [63]. Flavonoids regulate stomatal behavior, optimizing water use under drought conditions by modulating stomatal opening and closing, thereby improving water-use efficiency [64]. This regulation is closely linked to abscisic acid (ABA) signaling, which is crucial for maintaining plant growth and development under stress [65]. Furthermore, flavonoids, particularly flavones and flavonols, provide UV-B protection by accumulating in the epidermal layers of stems and leaves, where they act as a protective barrier against harmful radiations [66]. This photoprotective mechanism is vital for plants in high-solar-radiation environments, where UV-B exposure can cause significant cellular damage.

### 3.4. Tannins

Tannins are a group of phenolic compounds that act as repellents to pests and parasites, inhibiting their growth and reducing feeding activities [67]. These are classified into two main subgroups: condensed tannins and hydrolysable tannins, with molecular masses ranging from 600 to 3000 [68]. Their defense mechanisms include forming complexes with proteins, which deter herbivores and inhibit pathogen enzymes. Additionally, tannins are involved in regulating key defense signaling pathways, such as those mediated by jasmonic acid (JA) and salicylic acid (SA), which are essential for plant immune responses [69]. The unpleasant taste of tannins further deters herbivores, making them less palatable. Similarly, their antifungal properties are well-documented, such as the *PtMYB123* gene, regulating tannin biosynthesis and associating with systemic acquired resistance in plants. Tannin-rich extracts from *Acacia mearnsii* have demonstrated fungicidal activity, effectively inhibiting *Aspergillus niger* [70]. Furthermore, tannic acid and related phenolic compounds inhibit the activity of extracellular enzymes produced by pathogens, preventing the degradation of plant cell walls and protecting plant nutrients from depletion [71]. These findings highlight that tannins not only act as chemical deterrents against pests and pathogens but also modulate plant defense mechanisms through biochemical pathways, including JA and SA signaling.

### 3.5. Lignans and Lignin

Lignans are polyphenolic compounds predominantly found in seeds (e.g., flaxseeds) and other fibrous, phenolic-rich plant tissues [72]. Structurally, lignans are distinct from other secondary metabolites, characterized by specific carbon–carbon bonds and variations in carbon frameworks, oxygen positioning, and functional arrangements [73]. Lignans belong to the phytoestrogen family, which includes biologically active compounds, such as isoflavones, coumestans, and flavonoids. Their diverse structures contribute to a wide range of biological activities, including antioxidant properties that help plants manage oxidative stress during growth and development [74]. Lignans inhibit pathogen-derived degradative enzymes, such as cellulase, glucosidase, and laccase, thereby maintaining the integrity of plant cell walls and preventing microbial invasion [75].

Recent studies suggest that lignans modulate stress-related gene expression, enhancing the plant’s ability to cope with adverse environmental conditions [76]. Their ability to scavenge free radicals further protects plants from oxidative stress. Importantly, lignans regulate plant–pathogen interactions by reducing the activity of microbial enzymes, thereby protecting plants from pathogen-related damage [18]. These diverse biological activities highlight the importance of lignans in both biotic and abiotic stress responses, contributing significantly to plant resilience and adaptation [77].

Lignin, the second most abundant natural polymer after cellulose, plays a vital role in plant defense by enhancing cell wall strength and contributing to structural integrity. Derived from the phenylalanine/tyrosine pathway, lignin is a key metabolite involved in plant growth and defense [78]. Its biosynthesis involves the production of monomers, their transport, and polymerization by enzymes such as peroxidase (POD) and laccase (LAC) in the secondary cell wall [77]. As a structural component, lignin enhances cell wall rigidity, facilitates mineral transport, and protects against pests and pathogens. Additionally, lignin metabolism contributes to plant resistance and adaptation to environmental stress [12].

Lignin is synthesized through the combination of three phenolic compounds: coniferyl alcohol, sinapyl alcohol, and p-coumaryl alcohol [12,79]. As the second most abundant biopolymer after cellulose, lignin accounts for approximately 30% of the organic carbon in the biosphere. Its complex and branched structure serves as a physical barrier in the plant cell wall, blocking the invasion of pathogens and preventing damage from herbivores [80]. The chemical and physical properties of lignin make plant tissues tough and resistant to external damage, thereby strengthening the plant’s defense against pests and pathogens [12,81].

The defensive function of lignin is particularly evident in its ability to resist phytopathogenic fungi. Lignin forms a protective barrier around plant tissues, preventing fungal hyphae from penetrating the cell walls and spreading further. Studies have shown that lignin accumulation increases during pathogen attacks, indicating a protective response through a process known as lignification [82]. Furthermore, lignin is highly effective against fungal pathogens, such as *Diplodia pinea*, highlighting its antifungal potential compared to other phenolic compounds [83].

Beyond its role as a physical barrier, lignin contributes to other aspects of plant growth, such as cell wall rigidity, water transport, and overall plant hydrophobicity [84,85]. Its ability to resist herbivores, microbial pathogens, and environmental stresses underscores its multifunctionality in plant defense systems. Lignification is an adaptive response that makes plant tissues more indigestible to herbivores and limits pathogen growth during infection.

### 3.6. Stilbenes

Stilbenes are a subclass of secondary metabolites derived from the phenylpropanoid pathway. They are composed of a 14-carbon scaffold with two benzene rings connected by an ethylene bridge [21,86]. Stilbenes exist in two stereoisomeric forms, with the trans-isomer being the naturally occurring form in plants. These compounds play a crucial role in plant defense against both biotic and abiotic stresses [21].

Stilbenes exhibit direct toxic effects on bacteria and act as antioxidants, protecting plants from oxidative damage. They also influence fungal development and exhibit strong antifungal activity, likely due to their hydrophobic nature. For example, pterostilbene, a more mobile stilbene, spreads more effectively across the cytoplasmic membrane than resveratrol, which is less hydrophobic, showing enhanced antifungal properties [87,88]. Stilbene glucosides, produced in large quantities in the roots of *Polygonum cuspidatum*, enhance plant defense during pathogen stress [88]. Furthermore, stilbenes are elicited not only by pathogens but also by herbivores and other stressors, such as in the sapwood of plants [89].

### 3.7. Curcuminoids

Curcuminoids are polyphenolic compounds synthesized by plants, known for their potent antioxidant properties and significant role in plant defense. Approximately 1300 coumarins have been identified as secondary metabolites in plants, primarily contributing to plant defense and other functions [90,91]. These compounds are produced through type III polyketide synthases (PKSs) and consist of two phenylpropanoid components linked to a central moiety derived from malonyl-CoA [90,91]. Among curcuminoids, curcumin is the most well known and exhibits strong antioxidant and anti-inflammatory effects, protecting plants from oxidative stress and defending against microbial and fungal threats [91,92].

Curcumin has been shown to reduce cytokine levels, such as IL-1β and IL-6, and protect liver cells (e.g., hepatocytes, Kupffer cells, and endothelial cells) from apoptosis by inhibiting specific cellular signaling pathways [92]. The biosynthesis of curcumin is influenced by plant genotype, developmental stage, and exposure to stressors, such as pathogen attacks, which stimulate the production of curcuminoids to strengthen plant defenses [75,93]. Additionally, curcuminoids exhibit antiviral effects, including the reduction in viral RNA expression and virus titers, while their anti-inflammatory properties are mediated by their antioxidant activity [93]. These findings suggest that curcuminoids provide antioxidant and anti-inflammatory protection to plants, enhancing their defense against microbial, fungal, and viral stress, particularly in response to pathogen exposure.

### 3.8. Chitinases

Chitinases are enzymes that play a key role in plant defense against phytopathogenic fungi. These enzymes hydrolyze the β-1,4 linkages in the chitin of fungal cell walls, inhibiting fungal growth by breaking down hyphal tips [94,95]. Plants produce different types of chitinases, including secretory, cellular, and vacuolar chitinases, each serving specific functions in the defense process [96]. Secretory chitinases are involved in pathogenesis-related reactions and contribute to plant defense by interacting with fungal hyphae [95].

Genetic modifications have demonstrated that enhancing chitinase expression can increase resistance to fungal diseases in plants. For example, transgenic plants expressing chitinase DNA have shown improved resistance against fungal blight [96,97]. Moreover, chitinase activity is regulated by plant hormones, such as ethylene and jasmonate, which play a significant role in modulating local defense responses against pathogens [96]. In short, chitinases are vital enzymes to plant defense, breaking down fungal cell walls and providing resistance to fungal pathogens. Their activity is regulated by plant hormones in response to pathogen attacks, highlighting their importance in strengthening plant defenses.

### 3.9. Nitrogen- and Sulfur-Containing Secondary Metabolites

Approximately 20% of vascular plants produce nitrogen- and sulfur-containing secondary metabolites. These compounds, including alkaloids, cyanogenic glucosides, phytoalexins, non-protein amino acids, defensins, and lanine, are found across various plant groups, such as gymnosperms, monocots, and herbaceous plants [49,98]. Most of these metabolites are derived from simple amino acids, which serve as their precursors [98]. These nitrogen- and sulfur-containing metabolites are essential for plants to defend themselves against various biotic and abiotic stresses (Figure 3).

#### 3.9.1. Nitrogen-Containing Alkaloids

Alkaloids, nitrogen-containing secondary metabolites, are known for their antimicrobial properties. Specifically, polyamine alkaloids target Gram-negative bacteria by disrupting their external membrane and depolarizing the membranes of Gram-positive bacteria [99]. Phytoalexins, sulfur-containing metabolites, play a critical role in plant defense against fungal and bacterial pathogens [100,101]. These compounds also function in response to mechanical stress by reducing pathogen proliferation and inducing a hypersensitive response (HR), a form of programmed cell death [101]. Other sulfur-containing metabolites, such as lectins, defensins, and thionins, further bolster the plant’s immune system, providing resistance against microbial invaders [101].

#### 3.9.2. Sulfur-Containing Glucosinolates (GSLs)

Glucosinolates, found primarily in the mustard family (*Brassicaceae*), are activated when plant tissue is damaged, releasing toxic isothiocyanates through enzymatic breakdown by myrosinase [23,102]. These compounds serve as a defense against herbivores, such as the diamondback moth in cabbage plants [23,103]. Moreover, alkaloids, like vinblastine and vindoline, show increased levels in response to salinity and stress, contributing to plant adaptation and resilience [104]. In *Brassica napus* L., a major oil crop in North America and Europe, glucosinolate variation is influenced by age, developmental stage, and external factors such as mechanical damage, fungal infection, and insect attack. Treatment with methyl jasmonate or SA can enhance glucosinolate accumulation [104]. In *Arabidopsis*, glucosinolate biosynthesis begins with the conversion of amino acids to aldoxime, which conjugates with sulfur-donating cysteine and is cleaved by C-S lyase to form toxic thiohydroximate [12]. Glycosylation detoxifies thiohydroximate using uridine diphosphate glucose, producing desulfoglucosinolate via thiohydroximate glucosyltransferase.

#### 3.9.3. Sulfur-Containing Phytoalexins

Phytoalexins play a vital role in plant defense against bacterial and fungal diseases. Cruciferous phytoalexins, such as camalexin, brassinin, and rapalexin A, are indole alkaloids derived from (S)-tryptophan, often containing sulfur from cysteine [105]. These compounds are synthesized through pathways involving indole-3-acetonitrile (IAN) and glutathione, facilitated by glutathione S-transferase (GST) enzymes [100]. The accumulation of camalexin, regulated by cytochrome P450 enzymes (CYP79B2, CYP79B3), is crucial for biotic stress tolerance, as demonstrated in *Arabidopsis* pad3 mutants and AtABCG34-overexpressing plants [12]. Additionally, elemental sulfur, produced by crops like tomato, tobacco, and cotton in response to pathogens, is recognized as an inorganic phytoalexin.

## 4. Roles of Secondary Metabolites in Plant–Microbiome Interactions

Secondary metabolites, although not directly involved in growth or reproduction, play crucial roles in ecological interactions, particularly in plant–microbiome dynamics [60,106]. These compounds, including phenolics, alkaloids, terpenoids, and flavonoids, possess bioactive properties that modulate microbial populations, facilitate plant defense, and support environmental adaptation [107]. For instance, flavonoids promote nitrogen fixation in legumes, while terpenoids and alkaloids exhibit antimicrobial properties, suppressing harmful pathogens while encouraging beneficial microbes [60]. Thus, secondary metabolites not only defend plants but also shape the composition of plant-associated microbial communities, helping plants thrive in diverse environments.

### 4.1. Mechanisms of Interaction

Secondary metabolites mediate plant–microbe interactions through attraction, defense, and signaling mechanisms. They act as chemo-attractants for beneficial microbes. For example, phenolic acids create environments that promote the growth of beneficial microbes while inhibiting pathogens [60,108]. Flavonoids also promote the colonization of beneficial bacteria, improving the overall composition of the plant microbiome [60]. These metabolites defend plants by inhibiting pathogen growth and biofilm formation. For instance, certain metabolites disrupt pathogen signaling pathways, preventing the establishment of harmful microbes [18,109]. Coumarins and glucosinolates, in particular, exhibit potent antimicrobial properties [110].

Secondary metabolites, such as flavonoids, activate specific genes in microbes, fostering beneficial symbiotic relationships. For example, flavonoids activate nod genes in nitrogen-fixing bacteria, which is crucial for successful symbiosis [109]. This signaling promotes nutrient exchange between the plant and microbes, enhancing plant health and growth. While these interactions generally benefit plant health, some secondary metabolites may attract harmful microorganisms, underscoring the complexity of these relationships.

### 4.2. Bi-Directional Influence of Secondary Metabolites and Microbial Activity

The interaction between microbes and secondary metabolites is bi-directional. Microbial activity can enhance the production of secondary metabolites, bolstering plant defense mechanisms. For instance, the presence of arbuscular mycorrhizal fungi induces the production of terpenoids, which enhance the plant’s resistance to pathogens [21,111]. Similarly, microbial elicitors, such as beneficial bacteria, can trigger stress responses in plants, leading to the biosynthesis of alkaloids and other secondary metabolites that improve plant resilience [112]. The presence of beneficial microorganisms is key for evaluating terpenoid biosynthesis, which is an important factor in the plant’s ability to survive under biotic and abiotic stresses [21]. While the influence of microbes on secondary metabolite biosynthesis is mainly positive, it is important to note that the maximum production cycle may not always yield bio-improved novel products.

### 4.3. Role of Secondary Metabolites in Sustainable Agriculture

The relationship between secondary metabolites and plant–microbe interactions has significant implications for agricultural productivity and sustainability. Flavonoids, produced by legumes like *Medicago truncatula*, play a pivotal role in the nitrogen fixation process by signaling rhizobial bacteria to initiate root nodulation [113,114]. This interaction enhances soil productivity by reducing the need for synthetic fertilizers, supporting sustainable agricultural practices [57]. Additionally, phenolic compounds such as ferulic acid, secreted by wheat, promote the growth of beneficial rhizobacteria, like *Pseudomonas* and *Bacillus*, which produce antimicrobial substances to suppress soil-borne pathogens [114].

Phenolic compounds are also linked to improved disease resistance in wheat. Varieties with a higher total phenolic content (TPC), including flavonoid glycosides, exhibit enhanced resistance to diseases like stripe rust, contributing to higher yields [115]. Moreover, wheat cultivars with elevated polyphenol levels, such as Lincang Hulled Wheat (LHW), show improved resistance to pre-harvest sprouting, further supporting agricultural resilience [116]. These findings suggest that breeding crops with higher phenolic profiles can lead to more robust and productive agricultural systems. Secondary metabolites are integral to sustainable agriculture by enhancing plant resilience to stress and improving soil health, offering a pathway for reducing reliance on synthetic fertilizers and pesticides. As the research progresses, the potential of secondary metabolites in promoting agricultural sustainability becomes increasingly clear, highlighting the need for further exploration of these complex biochemical processes.

## 5. Expression and Manipulation of Gene Clusters for Secondary Metabolites Biosynthesis

**Figure 4:**
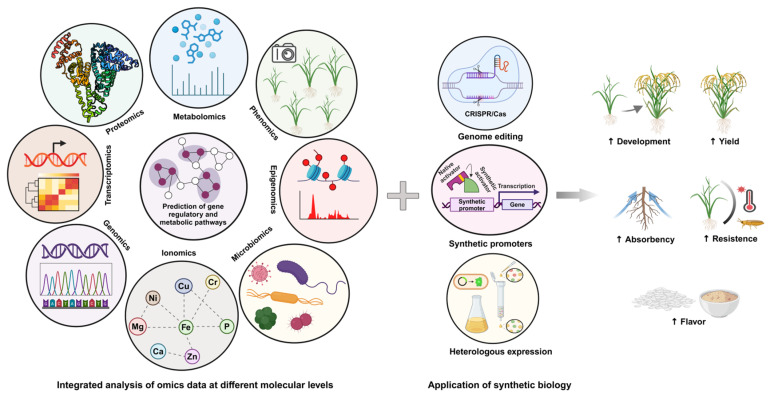
Strategies for studying the biosynthetic pathways of secondary metabolites. The intensification of climate change exacerbates the impact of environmental stress on crop productivity. Understanding the roles of primary and secondary metabolites in stress resistance mechanisms is crucial for developing crop varieties with improved stress tolerance, ensuring food security for an expanding global population. Advanced “omics” technologies, bioinformatics, and integrated molecular data analysis will provide deeper insights into secondary metabolite (SM) biosynthesis. Additionally, identifying the genetic basis of metabolite diversity in plants will enhance efforts to improve stress resilience. Genetic manipulation and overexpression of key genes in secondary metabolite biosynthetic pathways offer promising solutions to enhance plant tolerance to environmental stresses.

The biosynthesis of secondary metabolites in plants involves complex pathways regulated by multiple genes, often organized in biosynthetic gene clusters (BGCs). These pathways are difficult to study due to redundancy, regulatory complexity, and intricate networks [117]. To overcome these challenges, advanced expression strategies and genetic manipulation techniques are employed to enable the study and optimization of secondary metabolites biosynthesis (Figure 4). Regulatory networks involving transcription factors (e.g., MYB, WRKY, and NAC) and phytohormones (e.g., ABA, JA, and SA) modulate stress-responsive genes [118]. Epigenetic modifications (e.g., DNA methylation and histone changes) fine-tune gene expression for adaptive responses. Signal transduction pathways (e.g., ROS signaling and MAPK cascades) activate defense mechanisms against biotic and abiotic stresses [119].

Heterologous expression, where plant genes are expressed in microbial hosts, like *Escherichia coli*, *Saccharomyces cerevisiae*, and *Streptomyces* species, has become a key method for studying secondary metabolites biosynthesis. This approach bypasses the complexity of native plant systems, allowing the researchers to better understand metabolic pathways [117,120]. Using these hosts, which offer rapid growth rates and genetic tractability, plant genes are cloned and expressed in microbial systems. The researchers then study the resulting metabolites and elucidate biosynthetic pathways [120,121]. Bioinformatics tools and genome sequencing help identify BGCs, and subsequent experiments, such as precursor feeding, enable the validation of complete biosynthetic pathways [121]. Synthetic biology has further advanced heterologous expression by facilitating the modular design of biosynthetic pathways, improving metabolite yields, and enabling the discovery of novel SMs with potential applications in agriculture and medicine [122].

In addition to heterologous expression, manipulating BGCs and cryptic genes has proven to be a powerful strategy for optimizing secondary metabolite production [123]. Tools like CRISPR-Cas9 allow precise gene editing, which can induce targeted knockouts or modifications in plant DNA [123]. For example, CRISPR-Cas9 was used to mutate the HOS1 gene in *Arabidopsis thaliana*, impacting SM levels by altering gene expression and leading to changes in glucosinolate and flavonoid glycoside production [49]. Other transcription factors (TFs) like MYB and bHLH can be targeted to fine-tune SM biosynthesis under various stress conditions. RNA-based tools, such as RNA interference (RNAi) and transcription activator-like effector nucleases (TALENs), complement CRISPR by providing additional methods for regulating gene expression [124].

**Figure 5:**
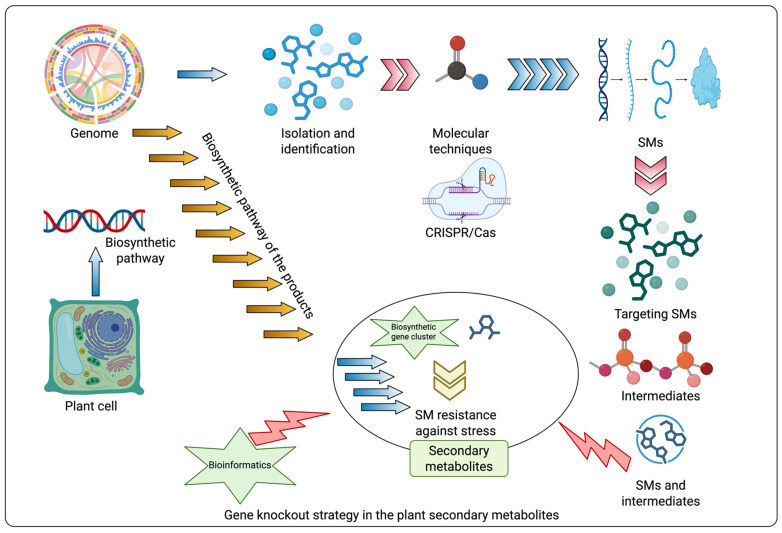
Mechanism of gene cluster biosynthetic pathways of secondary metabolites.

The integration of heterologous expression and advanced genetic tools, like CRISPR-Cas9 and RNAi, has revolutionized the study of SM biosynthesis (Figure 5). Genome sequencing has revealed numerous BGCs [123], encoding compounds such as terpenoids, phenolics, and non-ribosomal peptide synthetase (NRPS) products, opening new avenues for exploration [123]. Synthetic biology approaches further enhance the discovery and production of these metabolites, which can be used in stress tolerance and industrial applications. Together, these innovations have not only enhanced our understanding of plant stress responses, but also allowed the discovery and optimization of novel compounds for various applications.

## 6. Transcriptional Regulation of Secondary Metabolite Biosynthesis Under Biotic and Abiotic Stress Conditions

The biosynthesis of secondary metabolites under stress conditions is highly regulated by intricate genetic and transcriptional networks, involving key TFs, signaling pathways, and epigenetic modifications [125]. Key TF families, such as MYB, bHLH, WRKY, AP2/ERF, and NAC, regulate secondary metabolite biosynthesis by activating or repressing stress-responsive genes [126]. For example, MYB TFs regulate flavonoid and anthocyanin biosynthesis, with *AtMYB12* in *Arabidopsis* controlling flavonol production. bHLH TFs are involved in alkaloid biosynthesis, such as jasmonate-responsive bHLH factors in *Catharanthus roseus*, which control terpenoid indole alkaloid (TIA) pathways. WRKY TFs are essential for phenolic compound synthesis, with *AtWRKY18* in *Arabidopsis* regulating phytoalexin accumulation during pathogen attacks [127]. Secondary metabolite biosynthesis often requires combinatorial TF action. For instance, the MBW complex (MYB-bHLH-WD40) modulates anthocyanin synthesis, while MYC2 (bHLH) and ERF TFs cooperate in alkaloid biosynthesis [128].

### 6.1. Hormonal and Signal Transduction Pathways

Hormonal signaling pathways play a critical role in regulating secondary metabolite biosynthesis under stress conditions. JA-responsive genes, such as those in the JAZ-MYC module, regulate the production of alkaloids, terpenoids, and flavonoids in response to herbivore or pathogen attacks [129]. SA is involved in the biosynthesis of phenolic and sulfur-containing secondary metabolites, such as glucosinolates in *Brassicaceae*. ABA modulates stress-induced secondary metabolite pathways, particularly under drought or salt stress conditions [125]. Additionally, ethylene and brassinosteroids (BRs) synergistically regulate secondary metabolite biosynthesis in response to environmental cues.

### 6.2. Epigenetic Regulation and Chromatin Remodeling

Epigenetic regulation and chromatin remodeling are crucial for fine-tuning secondary metabolite biosynthesis under stress conditions. Histone modifications, such as acetylation (e.g., by HATs) and methylation (e.g., by HMTs), influence the activation of secondary metabolite biosynthetic genes [130]. For example, H3K9 acetylation enhances alkaloid biosynthetic gene expression in *Nicotiana*. DNA methylation of promoters of key biosynthetic genes, such as the phenylalanine ammonia-lyase (PAL) gene, alters phenylpropanoid biosynthesis [131]. Non-coding RNAs, such as miRNAs (e.g., miR156), regulate TFs involved in flavonoid biosynthesis and fine-tune metabolic responses.

Stress-specific regulatory modules further modulate secondary metabolite biosynthesis. Under biotic stress, such as pathogen or herbivore attacks, phytoalexin and glucosinolate biosynthesis are induced via WRKY and ERF TFs [132]. Under abiotic stress, such as drought, UV radiation, and temperature fluctuations, secondary metabolite pathways are modulated through ABA-responsive elements and ROS-mediated transcriptional control [133].

## 7. Biotechnological Advances in Engineering Secondary Metabolite Pathways

Advances in biotechnology, particularly in molecular biology, metabolic engineering, and synthetic biology, have enhanced our ability to manipulate secondary metabolite pathways [134,135]. Abiotic stresses, such as drought, salinity, and temperature extremes, often induce secondary metabolite production, helping plants adapt to challenging environments [135]. Engineering secondary metabolite pathways under abiotic stress conditions is now a major focus, offering opportunities to boost metabolite production and uncover stress-response mechanisms in plants [136]. These mechanisms include morphological, structural, molecular, biochemical, genetic, epigenetic, symbiotic, and microbial interactions [137]. For example, flavonoids and alkaloids play critical roles in stress responses, protecting against oxidative stress, enhancing drought and salinity tolerance, and defending against pathogens [138].

CRISPR/Cas9 technology enables the precise regulation of secondary metabolite production by enhancing or reducing the activity of specific genes. This has been demonstrated in plants like *Salvia miltiorrhiza* and *Medicago truncatula*, where gene modifications altered metabolite levels [139]. CRISPR-based approaches modulate various secondary metabolites, including flavonoids, alkaloids, and terpenoids, which are critical for stress adaptation. Recent advancements in systems biology allow the researchers to exploit abiotic stress to stimulate secondary metabolite biosynthesis [140]. Technologies such as transcriptomics, proteomics, and metabolomics provide insights into the cellular response to stress and guide the identification of key genes and proteins involved in secondary metabolite pathways [141]. Omics technologies enable the optimization of secondary metabolite production by analyzing how stress-induced signaling pathways activate biosynthetic gene clusters [142,143].

TFs are key to regulating secondary metabolite biosynthesis. For example, the overexpression of TFs, like MYB and WRKY, can enhance the production of metabolites under stress conditions [143]. These TFs coordinate multiple biosynthetic genes and help optimize secondary metabolite pathways, improving stress resistance in plants. By integrating transcriptomic, proteomic, and metabolomic data, the researchers can identify bottlenecks in metabolic pathways and design strategies to overcome them [144]. This approach has been applied to enhance antioxidant production by targeting the phenylpropanoid pathway under stress conditions.

Synthetic biology has enabled the transfer of stress-responsive secondary metabolite pathways into microbial hosts, like *Escherichia coli* and *Saccharomyces cerevisiae* [144,145]. CRISPR-based tools, including CRISPR activation (CRISPRa) and interference (CRISPRi), allow the fine-tuned control of gene expression to optimize secondary metabolite production [146]. Additionally, microbial consortia, where different strains cooperate to enhance secondary metabolite synthesis, offer an innovative approach to improving production efficiency [147].

Biotechnological advances in engineering secondary metabolite pathways have unlocked new possibilities for sustainable metabolite production. By utilizing tools like CRISPR, synthetic biology, and omics technologies, the researchers can harness stress responses to boost secondary metabolite biosynthesis and discover novel metabolites. These innovations hold promise for addressing challenges in medicine, agriculture, and environmental sustainability.

## 8. Conclusions and Prospect

Plants, as sessile organisms, face multiple biotic stresses, such as pathogen attacks and herbivore feeding, and abiotic stresses, including drought, salinity, and temperature, which threaten their survival and agricultural productivity. This review demonstrates that secondary metabolites (SMs) serve as central regulators in plant defense, enabling adaptation to these diverse challenges. Unlike primary metabolites that sustain basic cellular functions, SMs such as terpenes, flavonoids, phenolic compounds, and sulfur-containing phytoalexins act as frontline protectors, mitigating oxidative damage, deterring herbivores, and enhancing pathogen. For instance, stilbenes and curcuminoids combat microbial infections, while chitinases degrade fungal cell walls, collectively bolstering plant resilience. Moreover, SMs modulate physiological processes, like photosynthesis, stomatal regulation, and ion balance, as seen with flavonoids under drought stress. This multifaceted role is underpinned by intricate biosynthetic pathways (e.g., shikimic acid and mevalonic acid) and transcriptional networks involving MYB, WRKY, and hormonal signaling, which this review uniquely integrates across multi-stress contexts. Additionally, SMs shape plant–microbiome interactions and offer biotechnological potential through gene cluster manipulation, positioning them as key targets for sustainable agriculture amid climate change.

Despite these advances, critical gaps remain in understanding SM biosynthesis and application. How do specific stress signals (e.g., UV radiation vs. pathogen elicitors) differentially activate biosynthetic gene clusters for compounds like glucosinolates or alkaloids? What molecular mechanisms govern SM accumulation under combined stresses, a scenario increasingly relevant to climate change? Future research should prioritize elucidating these regulatory dynamics, building on transcriptional insights to map gene networks with precision. Multi-omics approaches with an integration of genome-wide association studies, comparative transcriptomics, targeted proteomics, and untargeted metabolomics can offer a powerful framework to address these issues [148]. For example, identifying genetic loci for tannin production under salinity or modeling terpenoid pathways in drought-stressed maize could reveal novel stress-responsive SMs and intermediates [149]. Systems biology can further construct predictive models of SM networks, enhancing the strategies outlined in Section 7 (e.g., CRISPR-based editing of MYB TFs in Salvia).

Practically, external SM supplementation (e.g., foliar curcumin application) and synthetic biology for in vitro production (e.g., microbial flavonoid synthesis) warrant a field-scale exploration to boost crop resilience. Breeding programs should target SM-rich traits such as flavonoid-enhanced wheat for rust resistance or glucosinolate-fortified *Brassica* for pest deterrence linked to microbiome benefits. These efforts, grounded in this comprehensive review, can transform the SM research into actionable solutions, ensuring food security in an era of high climatic changing conditions.

**Table 1:** List and role of secondary metabolites (SMs) in plants.

Name	Related Functions	Plant Specie	References
	**Terpenes**		
Monoterpenes	Chemical products secreted by plants are important against insect toxicity	*Chrysanthemum, cumin, pepper, mint, eucalyptus*	[150]
Diterpenes	Act as epithelium irritants and toxins to insects and mammals	*Codiaeum, Hura* *Phyllanthus*	[151]
Triterpenes	Triterpenes have some self-protective effects against insects by altering their development	*Higher plants* *Ferns and marine organisms*	[152]
Polyterpenes	Offer defense as a process for infection repair and as resistance to pests	*Bruce banner*	[153]
	**Phenolics**		
Phenolics flavonoids Coumarin Bioflavonoids Others	Flavanol content is significantly lower under the lower temperature treatment in pygmy smartweed	*Polygonum minus Huds.*	[154]
-	HT has little effect on seed phenolics, but reduces anthocyanins in the skin of grapes	*Vitis vinifera* L.	[155]
-	Monoterpenes and sesquiterpenes increase in thyme in response to DS	*Artemisia annua* L.	[156]
-	Monosubstituted flavanols increase under UVB Flavanols are unaffected; supplemental UVB also increases tannins in some species	*Tomato*	[54]
	**Nitrogen-containing SMs**		
Alkaloids Cyanogenic glycosides Non-Protein Amino Acid	Cause signaling molecule to trigger flavonoid biosynthesis under lower temperatures	*Apple* (*Malus* sp.)	[54]
-	Increased light may have negative consequences on SM production in sensitive plants. Longer photoperiod	*Ocimum basilicum* L.	[157]
-	Plants have more cyanogenic glycosides; variability also observed in alkaloids, which increases in the shade in evergreen tropical trees	*Tabernaemontana pachysiphon Stapf*	[54]
-	*Arabidopsis* mutants lacking flavonoids; production mechanisms are hypersensitive to UVB radiation; flavonoid production is tolerant to typically lethal UVB levels	*Arabidopsis thaliana*	[158]
	**Sulfur-containing SMs**		
Glutathione	GSH acts as a growth regulator and during stress it acts as an antioxidant, strengthening the defense system of the plants	*Spinach* *Avocados* *Okara*	[159]
Glucosinolate GLS	Plays a role in defense by poisoning herbivore insects during damage and as a feeding repellent	*Mustar Allium allylcysd plant*	[160]
Phytoalexins	This is a common defense mechanism against insect pests in numerous plants	*Grapevine Vitis vinifera*	[161]
Defensins, thionins, and lectins	Defensins, thionine, and lectins are stimulated by numerous stresses and show resistance against them	Circulatory white blood cells and tissue cells, wheat, corn, and tomato	[162]
	**Stilbenes**		
Resveratrol and pterostilbene)	Increased stilbene accumulation, greater with UV-C compared to fungal inoculum, and shows resistance	*Vitis vinifera* cvs. *Alphonse Lavallée, Dan Ben-Hanna*	[163]
anthocyanins; flavonoids; hydroxycinnamic acids Napoleon	Increased stilbene accumulation, greater with UV-C compared to UV-B (3- and 2-fold, respectively), and shows resistance	*V. vinifera* cv. *Sangiovese*	[163]
Stilbenes	Downregulation of STS expression under both low and high temperatures, upregulation of STS expression in response to CuSO4, and shows resistance	*V. vinifera* cv. *Cabernet Sauvignon*	[163]
Mono-glucosylated derivative resveratrol (trans- and cis-piceid and trans- and cis-resveratroloside)	Increase in trans-resveratrol endogenous accumulation and decreased release into the culture medium Glucosides show response to stress	*V. vinifera* cv. *Barbera*	[163]
	**Curcuminoids**		
Curcumin	Physical and chemical defense against pathogens as well as other stresses	*Curcuma longa.* L.	[164]
Curcumin/bisdemethoxycurcumin	Volatile compound shows antibacterial mechanism against a wide distribution of Gram-positive bacteria,	*Curcuma longa.* L.	[165]
Demethoxycurcumin	which have antipathogenic action against fungi, bacteria, and other pathogen agents	*Turmeric*	[166]
	**Chitinases**		
Maize chitinase 2 gene	Secondary metabolites considered as molecular targets of selection in plant–pathogen interactions.	*Transgenic maize plant*	[167]
Chitinase I gene	Inhibits phytopathogenic fungi *A. solani*, *R. solani*, *F.* spp., and *V. dahliae*	*Hordeum vulgare cultivar, Haider-93*	[96]
Rice class I chitinase gene (Rchit)	Resistance against late leaf spot, rust disease, and *A. flavus* infection	*Oryza sativa (Rice)*	[168]
Tobacco osmotin (ap24) and rice chitinase (chi 11) gene	Reduce sheath blight disease caused by *R. solani*	*Nicotiana* sp. *(Tobacco) and Oryza sativa (Rice)*	[169]
Rice chitinase-3 gene	Resistance against leaf spot in peanut by *Cercospora arachidicola*	*Oryza sativa (Rice)*	[170]
	**Peroxidase**		
Glutathione peroxidase	Causes a reduction in the substrate to convert H_2_O_2_ hydroperoxides into water or oxygen, and shows resistance	*Nicotiana* sp. *(Tobacco)*	[96]
Horseradish peroxidase	Plants have adopted peroxidase systems to show resistance against numerous stresses	*Armoracia rusticana*	[171]
Cytochrome c peroxidase	These enzymes use peroxides as an electron acceptor for a reduction in oxidative damage due to stress in plants	*Yeast*	[172]
Myeloperoxidase	Includes plant immune responses to biotic stresses	*Spinach*	[173]

## Data Availability

No new data were created or analyzed in this study.
