# Correction: Khan et al. Plant Secondary Metabolites—Central Regulators Against Abiotic and Biotic Stresses. *Metabolites* 2025, *15*, 276

**DOI:** 10.3390/metabo16030154

**Published:** 2026-02-26

**Authors:** Ameer Khan, Farah Kanwal, Sana Ullah, Muhammad Fahad, Leeza Tariq, Muhammad Tanveer Altaf, Asad Riaz, Guoping Zhang

**Affiliations:** 1 Department of Agronomy, College of Agriculture and Biotechnology, Zhejiang University, Zijingang Campus, Hangzhou 310029, China; 12116133@zju.edu.cn; 2 National Key Laboratory for Tropical Crop Breeding, School of Breeding and Multiplication (Sanya Institute of Breeding and Multiplication), Hainan University, Sanya 572025, China; farah@hainanu.edu.au; 3 Department of Plant Breeding and Genetics, University of Agriculture, Faisalabad 38000, Pakistan; sanaullahpbg1@gmail.com; 4 Zhejiang Provincial Key Laboratory of Crop Genetic Resources, College of Agriculture and Biotechnology, Zhejiang University, Hangzhou 310058, China; muhammadfahad@zju.edu.cn; 5 National Key Laboratory for Rice Biology and Breeding, Institute of Biotechnology, Zhejiang University, Hangzhou 310058, China; leeza_tariq@zju.edu.cn; 6 Department of Field Crops, Faculty of Agriculture, Recep Tayyip Erdoğan University, Pazar, Rize 53300, Turkey; muhammadtanveer.altaf@erdogan.edu.tr; 7 Queensland Alliance for Agriculture and Food Innovation, The University of Queensland, Brisbane, QLD 4072, Australia

The authors would like to make the following correction to their published paper [1]. The change is as follows: Reference [157] in the original publication is incorrectly cited and needs to be removed, along with the content associated with this reference in Table 1. The corrected Table 1. List and role of secondary metabolites (SMs) in plants appears below:

157. Mukherjee, S.; Kutty, N.N.; Bera, P.; Mitra, A. Impact of light and sucrose supplementation on cellular differentiation, metabolic shift and modulation of gene expression in hairy roots of Daucus carota. *Plant Cell Tissue Organ Cult. (PCTOC)* **2019**, *136*, 383–397.

The authors state that the scientific conclusions are unaffected. This correction was approved by the Academic Editor. The original publication has also been updated.

## Figures and Tables

**Table 1:** List and role of secondary metabolites (SMs) in plants.

Name	Related Functions	Plant Specie	References
	**Terpenes**		
Monoterpenes	Chemical products secreted by plants are important against insect toxicity	*Chrysanthemum, cumin, pepper, mint, eucalyptus*	[150]
Diterpenes	Act as epithelium irritants and toxins to insects and mammals	*Codiaeum, Hura* *Phyllanthus*	[151]
Triterpenes	Triterpenes have some self-protective effects against insects by altering their development	*Higher plants* *Ferns and marine organisms*	[152]
Polyterpenes	Offer defense as a process for infection repair and as resistance to pests	*Bruce banner*	[153]
	**Phenolics**		
Phenolics flavonoids Coumarin Bioflavonoids Others	Flavanol content is significantly lower under the lower temperature treatment in pygmy smartweed	*Polygonum minus Huds.*	[154]
-	HT has little effect on seed phenolics, but reduces anthocyanins in the skin of grapes	*Vitis vinifera* L.	[155]
-	Monoterpenes and sesquiterpenes increase in thyme in response to DS	*Artemisia annua* L.	[156]
-	Monosubstituted flavanols increase under UVB Flavanols are unaffected; supplemental UVB also increases tannins in some species	*Tomato*	[54]
	**Nitrogen-containing SMs**		
Alkaloids Cyanogenic glycosides Non-Protein Amino Acid	Cause signaling molecule to trigger flavonoid biosynthesis under lower temperatures	*Apple* (*Malus* sp.)	[54]
-	Increased light may have negative consequences on SM production in sensitive plants. Longer photoperiod	*Ocimum basilicum* L.	[157]
-	Plants have more cyanogenic glycosides; variability also observed in alkaloids, which increases in the shade in evergreen tropical trees	*Tabernaemontana pachysiphon Stapf*	[54]
-	*Arabidopsis* mutants lacking flavonoids; production mechanisms are hypersensitive to UVB radiation; flavonoid production is tolerant to typically lethal UVB levels	*Arabidopsis thaliana*	[158]
	**Sulfur-containing SMs**		
Glutathione	GSH acts as a growth regulator and during stress it acts as an antioxidant, strengthening the defense system of the plants	*Spinach* *Avocados* *Okara*	[159]
Glucosinolate GLS	Plays a role in defense by poisoning herbivore insects during damage and as a feeding repellent	*Mustar Allium allylcysd plant*	[160]
Phytoalexins	This is a common defense mechanism against insect pests in numerous plants	*Grapevine Vitis vinifera*	[161]
Defensins, thionins, and lectins	Defensins, thionine, and lectins are stimulated by numerous stresses and show resistance against them	Circulatory white blood cells and tissue cells, wheat, corn, and tomato	[162]
	**Stilbenes**		
Resveratrol and pterostilbene)	Increased stilbene accumulation, greater with UV-C compared to fungal inoculum, and shows resistance	*Vitis vinifera* cvs. *Alphonse Lavallée, Dan Ben-Hanna*	[163]
anthocyanins; flavonoids; hydroxycinnamic acids Napoleon	Increased stilbene accumulation, greater with UV-C compared to UV-B (3- and 2-fold, respectively), and shows resistance	*V. vinifera* cv. *Sangiovese*	[163]
Stilbenes	Downregulation of STS expression under both low and high temperatures, upregulation of STS expression in response to CuSO4, and shows resistance	*V. vinifera* cv. *Cabernet Sauvignon*	[163]
Mono-glucosylated derivative resveratrol (trans- and cis-piceid and trans- and cis-resveratroloside)	Increase in trans-resveratrol endogenous accumulation and decreased release into the culture medium Glucosides show response to stress	*V. vinifera* cv. *Barbera*	[163]
	**Curcuminoids**		
Curcumin	Physical and chemical defense against pathogens as well as other stresses	*Curcuma longa.* L.	[164]
Curcumin/bisdemethoxycurcumin	Volatile compound shows antibacterial mechanism against a wide distribution of Gram-positive bacteria,	*Curcuma longa.* L.	[165]
Demethoxycurcumin	which have antipathogenic action against fungi, bacteria, and other pathogen agents	*Turmeric*	[166]
	**Chitinases**		
Maize chitinase 2 gene	Secondary metabolites considered as molecular targets of selection in plant–pathogen interactions.	*Transgenic maize plant*	[167]
Chitinase I gene	Inhibits phytopathogenic fungi *A. solani*, *R. solani*, *F.* spp., and *V. dahliae*	*Hordeum vulgare cultivar, Haider-93*	[96]
Rice class I chitinase gene (Rchit)	Resistance against late leaf spot, rust disease, and *A. flavus* infection	*Oryza sativa (Rice)*	[168]
Tobacco osmotin (ap24) and rice chitinase (chi 11) gene	Reduce sheath blight disease caused by *R. solani*	*Nicotiana* sp. *(Tobacco) and Oryza sativa (Rice)*	[169]
Rice chitinase-3 gene	Resistance against leaf spot in peanut by *Cercospora arachidicola*	*Oryza sativa (Rice)*	[170]
	**Peroxidase**		
Glutathione peroxidase	Causes a reduction in the substrate to convert H_2_O_2_ hydroperoxides into water or oxygen, and shows resistance	*Nicotiana* sp. *(Tobacco)*	[96]
Horseradish peroxidase	Plants have adopted peroxidase systems to show resistance against numerous stresses	*Armoracia rusticana*	[171]
Cytochrome c peroxidase	These enzymes use peroxides as an electron acceptor for a reduction in oxidative damage due to stress in plants	*Yeast*	[172]
Myeloperoxidase	Includes plant immune responses to biotic stresses	*Spinach*	[173]
